# Complement C4 Deficiency – A Plausible Risk Factor for Non-Tuberculous Mycobacteria (NTM) Infection in Apparently Immunocompetent Patients

**DOI:** 10.1371/journal.pone.0091450

**Published:** 2014-03-17

**Authors:** Hannele Kotilainen, Marja-Liisa Lokki, Riitta Paakkanen, Mikko Seppänen, Pentti Tukiainen, Seppo Meri, Tuija Poussa, Jussi Eskola, Ville Valtonen, Asko Järvinen

**Affiliations:** 1 Division of Infectious Diseases, Department of Medicine, Helsinki University Central Hospital, Helsinki, Finland; 2 Transplantation Laboratory, Haartman Institute, University of Helsinki, Helsinki, Finland; 3 Division of Cardiology, Department of Medicine, Helsinki University Central Hospital, Helsinki, Finland; 4 Division of Lung Diseases, Department of Medicine, Helsinki University Central Hospital, Helsinki, Finland; 5 Department of Bacteriology and Immunology, Haartman Institute, University of Helsinki, Helsinki, Finland; 6 STAT-Consulting, Nokia, Finland; 7 Mycobacteriology Unit, Helsinki University Central Hospital Laboratory, Helsinki, Finland; Oxford University, United Kingdom

## Abstract

**Background:**

Non-tuberculous mycobacteria (NTM) are ubiquitous in the environment and they infect mainly persons with underlying pulmonary diseases but also previously healthy elderly women. Defects in host resistance that lead to pulmonary infections by NTM are relatively unknown. A few genetic defects have been associated with both pulmonary and disseminated mycobacterial infections. Rare disseminated NTM infections have been associated with genetic defects in T-cell mediated immunity and in cytokine signaling in families. We investigated whether there was an association between NTM infections and deficiencies of complement components *C4A* or *C4B* that are encoded by major histocompatibility complex (MHC).

**Methods:**

50 adult patients with a positive NTM culture with symptoms and findings of a NTM disease were recruited. Patients' clinical history was collected and symptoms and clinical findings were categorized according to 2007 diagnostic criteria of The American Thoracic Society (ATS). To investigate the deficiencies of complement, *C4A* and *C4B* gene copy numbers and phenotype frequencies of the C4 allotypes were analyzed. Unselected, healthy, 149 Finnish adults were used as controls.

**Results:**

NTM patients had more often *C4* deficiencies (*C4A* or *C4B*) than controls (36/50 [72%] vs 83/149 [56%], OR = 2.05, 95%CI = 1.019–4.105, p = 0.042). *C4* deficiencies for female NTM patients were more common than for controls (29/36 [81%] vs 55/100 [55%], OR = 3.39, 95% CI = 1.358–8.460, p = 0.007). C4 deficiences seemed not to be related to any specific underlying disease or C4 phenotype.

**Conclusions:**

*C4* deficiency may be a risk factor for NTM infection in especially elderly female patients.

## Introduction

Non-tuberculous mycobacterial (NTM) infections have been related to underlying pulmonary diseases [1], [2], [3]. In recent studies, however, up to two-thirds of NTM infections were found in patients without predisposing lung diseases with a predominance of elderly female patients [4], [5], [6], [7]. Host-related causal factors of pulmonary and extra pulmonary NTM infections in elderly immunocompetent patients are mostly unknown [8], [9], [10]. Recently, reduced nasal nitric oxide production, reduced ciliary beat frequency and abnormal toll like receptor responses were observed in pulmonary NTM patients [11]. Familial clustering and genetic risk factors for pulmonary NTM diseases have been suggested in earlier studies [12], [13], [14], [15]. The rare disseminated NTM diseases have been reported in the families with a few mutations in interferon- γ (INF-γ) and interleukin (IL-12) signaling [15], [16], [17], [18], [19]. However, these defects in INF-γ/IL-12 production have not been found among pulmonary NTM patients [19], [20]. Acquired neutralizing auto-antibodies against INF-γ have been associated with multiple opportunistic infections, which include disseminated NTM [21]. Therefore, the pathogenesis of the pulmonary NTM obviously differs from those of the disseminated NTM infections. Clinically, the pulmonary NTM patients are diagnosed late. Consequently, the patients will have already developed structural defects by the time diagnosis is made [10].

Genetic susceptibility to pulmonary tuberculosis has been associated with major histocompatibility complex (MHC) class I, II and III regions on chromosome 6p21.3 [19], [22]. Complement genes *C4 (C4A, C4B), C2*, and *FB* are located in the MHC class III region between the class I (HLA-A, -C, -B) and the class II (HLA-DR, -DQ, -DP) [19], [22]. Pulmonary tuberculosis has been associated to human leukocyte antigen (HLA) class I and II genes in several populations with variable and moderate susceptibilities [23], [24], [25], [26], [27]. MHC complement genes *C2, C4*, and factor *B (FB)*, and *C3* have been studied in an Indian population showing increased frequencies of complete *C4A* deficiency, BF*FA and C3*F in patients with pulmonary tuberculosis compared with healthy individuals [22].

The complement system is involved both in innate and acquired immune responses consisting of a triggered-enzyme cascade activated by immunoglobulins, mainly by IgG1 [28]. Activated complement proteins bind covalently to pathogens, thereby opsonizing them prior to the antigen's engulfment by macrophages, which in turn bear the receptors for the complement factors [28]. However, the capsular lipid layer of the mycobacteria makes them resistant to the host's defense mechanisms, and thus the mycobacteria can evade phagocytosis and lysosomes [29]. The *Mycobacterium tuberculosis* (MTB) survives and multiplies within macrophages and adapt to the hostile environment [30], [31]. *Mycobacterium bovis* (BCG) has been shown to activate all three complement pathways and the important role of the INF-γ pathway in host defence has been revealed [20]. The defects of the complement genes will produce quantitatively less active complement proteins, which will thus hamper the opsonization and positive feedback of IL-12/IFN- γ of the macrophages [30]. Possibly, the opportunistic NTM may also use this mechanism to evade the host defense.

We evaluated the deficiencies of complement components *C4A* and *C4B* in patients with a NTM infection. Patients were evaluated according to American Thoracic Society (ATS) 2007 criteria [32]. We compared the frequencies of *C4* deficiencies in the NTM patients with those of the unselected healthy Finnish adult controls. We found increased frequencies of C4 deficiencies to be a plausible risk factor for NTM infection.

## Materials and Methods

### Study populations

In total, there were 257 patients with a NTM isolation in the patient recruitment area of the study hospital during the study period. These patients were admitted to the Division of Lung Diseases or to the Division of Infectious Diseases, at Helsinki University Central Hospital between August 2004 and December 2009 due to symptoms of a NTM disease. Out of admitted patients we recruited 52 voluntary patients, aged over 18 years, who were culture-positive for a non-tuberculous mycobacterial infection. 50 NTM patients gave their written informed consent after information was given face-to-face. Two patients were excluded due to missing consent. There were no cases with a family history of a mycobacterial disease among the recruited patients. Peripheral blood samples of 35 ml were drawn from patients for allotypic and genetic analysis of complement *C4* in an average within one month of admission to hospital before any antimycobacterial therapy. According to the study protocol patients who were pregnant, handicapped, prisoners, under 18 years of age, conscripts and patients with HIV or CVI (common variable immunodeficiency) were excluded from the study. The control cohort (*n* = 149) consisted of healthy, voluntary blood donors, 100 females and 49 males, had been recruited in a health survey of the Helsinki region [33]. The Ethics Committee of the Department of Medicine, Hospital District of Helsinki and Uusimaa approved the study protocol March 1, 2004.

### Case definitions

The NTM infection patients were categorized according to ATS criteria 2007 [32] in order to reveal how many fulfilled the ATS diagnosis for NTM disease. According to these diagnostic criteria, a patient should have NTM isolated in at least two sputum cultures, or one positive culture from a bronchoscopic sample (by lavage or by brush) or from a lung biopsy to fulfill the microbiological criteria. One positive culture from a skin or lymphatic tissue biopsy fulfilled the microbiological criteria for extra pulmonary disease. Radiological criteria were fulfilled when nodular or cavitary opacities were found on chest radiographs or computer tomography (CT), or when bronchiectasis with multiple small nodules were found by CT. The numbers of subsequent positive mycobacterial smears and cultures and the site of sampling were also recorded.

Demographic characteristics of the patients including age, sex, and occupation, were obtained from the patient records. Each patient's previous medical history was reviewed, and concomitant diseases were classified according to McCabe and Jackson [34] as follows: (1) healthy, i.e. no other disease, (2) chronic nonfatal disease, (3) ultimately fatal disease with an expected life expectancy of 5 years maximum, and (4) rapidly fatal disease with expected survival of no more than 6 months.

In particular, information on previous pulmonary diseases, such as bronchiectasis, COPD, pulmonary fibrosis, prior tuberculosis, asthma, pulmonary malignancies and pneumonias, were retrieved. Signs, symptoms, laboratory findings and radiological examinations were reviewed at the time of recruitment or closest +/−6 months. Radiological findings on chest X-ray, CT and high-resolution computed tomography (HRCT) were classified based on the clinical radiologists statement as infiltrates, nodules, cavities or bronchiectasis according to the ATS criteria 2007 [32]. The anatomical localization of the findings for each lobe was recorded separately or as diffuse findings in both lungs. Current (within 6 months of recruitment) immunosuppressive treatments were reviewed.

All 50 NTM patients fulfilled the ATS 2007 criteria for symptoms and all but one fulfilled the bacteriological criteria as well. The sole exception was a male patient with pulmonary cancer. That patient did not fulfill bacteriological criteria and he had bilateral nodules in his lungs that could be classified as either belonging to NTM or to the malignancy, which made fulfillment of the radiological criteria uncertain. This patient presented with a sputum smear positive finding, which included other NTM, according to PCR, but the culture remained negative. The patient died soon after without further examinations being made. Radiological criteria were fulfilled for 29/44 (66%) with pulmonary NTM. Fifteen patients 15/44 (34%) did not fulfill the radiological criteria due to cancer or COPD findings without nodules, cavities or bronchiectasis. However, all except one patient repeatedly gave positive NTM sputum samples over the time of recruitment (closest +/− one year). All six patients with extra pulmonary NTM fulfilled the ATS 2007 criteria.

### Laboratory methods

All samples for mycobacterial staining except blood samples were stained with auramine-O-fluorochrome dye and examined microscopically for the presence of acid-fast bacilli (AFB). Cultures that were positive for acid-fast bacilli were identified by DNA strip assays (GenoType Mycobacterium CM/AS, Hain Lifescience, Nehren, Germany). *Mycobacterium avium comple*x (MAC), *M. avium*, and *Mycobacterium intracellulare* were all classified as MAC.

The numbers of *C4A* and *C4B* genes and allotypes of C4A and C4B proteins were determined in the HLA Laboratory, which is a division of the Transplantation Laboratory at the Haartman Institute, University of Helsinki, as previously published [35]. The HLA Laboratory has international EFI-accreditation (European Federation for Immunogenetics). All samples were kept frozen at −70°C. CT insertion, a *C4*-silencing mutation, was reduced from the amount of *C4A* genes. *C4* deficiency was defined as the presence of less than two copies of *C4A* or *C4B*.

### Statistical methods

All statistical tests were performed using IBM SPSS statistics software (version 20, NY). Differences in proportions between groups were tested by the Chi-square test or by Fisher's exact two-tailed test, according to which was appropriate. Continuous variables that were not normally distributed were transformed as such by using logarithmic transformation. The continuous variables were compared using the Student's t-test. The Mantel-Haenszel method and the Breslow-Day test were used to test the homogeneity of the odds ratios between males and females. The sample size calculation was based on the prevalence previously reported 58% prevalence of C4 deficiency in healthy controls and an assumption of C4 deficiency in 80% of NTM patients [33]. A two group c^2^ test with a 0,05 two-sided significance level will have 80% power to detect the difference between these prevalences (odds ratio of 2,90) resulted in sample sizes are of 47 NTM patients and 141 healthy controls, respectively. P -values of <0.05 were considered statistically significant.

## Results

### Patient characteristics and mycobacterial strains

All NTM patients were of European descent. Forty-four patients had pulmonary NTM infection whereas six patients had extra pulmonary NTM infection. There was a clear female dominance among the NTM patients (36/50 [72%], Table 1). Notably, the NTM patients had a high incidence of ultimately or rapidly fatal underlying diseases (McCabe classes III–IV, 18/50 [36%] and a high incidence of bronchiectasis (15/50 [30%], Table 1).

**Table 1:** Characteristics,underlying diseases and laboratory findings of 50 patients with a non-tuberculous mycobacteria (NTM).

	NTM	
	n = 50	
	n	(%)
Age median (IQR) yr	65	(17)
Female	36	(72)
Pulmonary mycobacterial infection	44	(88)
Mycobacterial infection in lymph nodes	1	(2)
Cutaneous mycobacterial infection	5	(10)
Underlying diseases^a^		
Healthy or non-fatal diseases(1–2)	32	(64)
Ultimately or rapidly fatal diseases(3–4)	18	(36)
Underlying pulmonary diseases		
Bronchiectasis	15	(30)
COPD	13	(26)
Prior tuberculosis	3	(6)
Asbestosis	2	(4)
Asthma	4	(8)
Pulmonary or other malignancy	6	(12)
No previous pulmonary diseases	16	(32)
Rheumatoid arthritis	8	(16)
Other autoimmune disease	3	(6)
Never smokers	27	(54)
Current or ex-smokers	23	(46)
Laboratory finfindings		
ESR median (IQR) mm/h	18.5	(30)
CRP median (IQR) mg/l	6.5	(16)

Values are expressed as number (%) of patients with valid information unless otherwise stated.

a Underlying diseases classified according to the criteria of the McCabe classification:

1) healthy i.e. no other diseases.

2) non-fatal chronic diseases.

3) ultimately fatal diseases with expected life expectancy of maximally 5 years.

4) rapidly fatal diseases with expected survival for no more than 6 months.

MAC comprised 36/50 (72%) of all NTM isolations. Rapidly growing mycobacteriua (*M. fortuitum, M. chelonae, M. abscessus*) isolations accounted for 12% (6/50) isolations. Moreover, three patients had *M. malmoense* and two patients had *M. xenopi*. One patient had *M. marinum* and two patients other NTM. No patients had concomitant NTM and *M.tuberculosis* (MTB) infections.

### Complement analyses

*C4* deficiency was more prevalent in the NTM patients than in the healthy controls (36/50 [72%] vs 83/149 [56%], OR = 2.05, 95% CI = 1.019–4.105, p = 0.042, Table 2). Deficiencies of *C4A* and *C4B* were assessed individually but no statistically significant differences between the NTM and healthy patients were found (Table 2).

**Table 2:** Genotypic *C4* deficiencies in patients with non-tuberculous mycobacteria (NTM) and in healthy control population (H).

	NTM		H		NTM vs H	
	n = 50		n = 149			
Type of deficiency	n	(%)	n	(%)	OR (95% CI)	*p* a
*C4A*<2	13	(26)	24	(16)	1.83 (0.85–3.95)	0.120
*C4B*<2	25	(50)	61	(41)	1.44 (0.76–2.75)	0.263
*C4A* or *C4B*<2	36^b^	(72)	83	(56)	2.05 (1.019–4.105)	0.042
*C4*<4	22	(44)	56	(38)	1.30 (0.68–2.50)	0.421

Values are expressed as number (%) of patients, unless otherwise stated.

Abbreviations:

OR = odds ratio,

CI = confidence interval.

a p values for differences between groups.

b Two patients had both *C4A*<2 and *C4B*<2 deficiency, one patient had a total *C4A* deficiency.

The majority of the NTM patients were female; consequently we assessed the *C4* deficiencies in females only. We compared females in the healthy control group with females in the NTM patient group (Table 3). Deficiency of *C4* occurred more often in the female NTM patients than in the healthy female controls (29/36 [81%] vs 55/100 [55%], OR = 3.390, 95% CI = 1.358–8.460, p = 0.007, Table 3). There was a tendency for more common deficiency of both C4B and C4A in the females NTM patients as compared to female controls but these differences did not reach statistical significance (Table 3). In the subgroup analyses no difference in C4 deficiency was observed between male NTM and male control patients (7/14 [50%] vs 28/49 [57%], OR = 0.75, 95% CI = 0.23–2.47, p = 0.045) The subgroup analyses were justified as the Breslow-Day test found evidence of interaction between sex and infection (NTM vs. Healthy) with respect to type of deficiency (p = 0.108 for C4B<2 and p = 0.045 for C4Aor C4B<2). However, the number of men was too low for reliable analysis (n = 14 in NTM and n = 49 in H).

**Table 3:** Type of genotypic *C4* deficiency in female non-tuberculous mycobacteria (NTM) patients compared with healthy female control population (H).

	NTM female		H female		NTM female vs H female	
	n = 36		n = 100			
Type of deficiency	n	(%)	n	(%)	OR (95% CI)	p^a^
C4A<2	11^b^	(31)	17	(17)	2.148 (0.891–5.181)	0.085
C4B<2	20	(56)	38	(38)	2.04(0.94–4.41)	0.068
C4A or C4B<2	29^c^	(81)	55	(55)	3.390 (1.358–8.460)	0.007

Values are expressed as number (%) of patients, unless otherwise stated.

Abbreviations:

OR = odds ratio,

CI = confidence interval.

a p values for differences between groups.

b One female patient had a total *C4A* deficiency.

c Two female patients had both *C4A*<2 and *C4B*<2 deficiency.

Next, we specifically investigated whether a *C4* deficiency was associated with different patient characteristics of the NTM patients (Table 4). Either *C4A* or *C4B* deficiency was observed in 31/44 (70%) of the NTM patients with the pulmonary NTM infection. A *C4* deficiency was found in 22/29 (76%) of those pulmonary NTM patients who met all ATS 2007 criteria. All five patients with a cutaneous NTM infection met the ATS 2007 criteria and they all had *C4B* deficiency. *C4* deficiency was found in 67% of the patients who did not meet the radiological criteria, but who repeatedly gave a positive NTM sputum culture (Table 4).

**Table 4:** The clinical manifestations in 50 non-tuberculous mycobacteria (NTM) patients with a C4 deficiency.

		Underlying diseases^a^		NTM infection localization			ATS 20007 NTM criteria fullfillment			Underlying pulmonary diseases			
	Female	Healthy or non fatal disease (1–2)	Ultimately or rapidly fatal disease (3–4)	Pulmonary	Lymph node	Cutaneous	Symptoms and microbiological criteria fulfilled in pulmonary infection	Symptoms, microbiologcal and radiographic criteria fulfilled in pulmonary infection	Radiographic criteria not fulfilled in pulmonary infection	Bronchiectasies	COPD or asthma	No previous pulmonary diseases,	Rheumatic arthritis Autoimmundiseases
	n = 36	n = 32	n = 18	n = 44	n = 1	n = 5	n = 49	n = 29	n = 15	n = 15	n = 17	n = 16	n = 11
C4 deficiency							number of patients (%)						
C4 A<2	11 (31)	10 (31)	3 (17)	13 (30)	0 (0)	0 (0)	13 (27)	10 (34)	3 (20)	6 (40)	3 (18)	3 (19)	1 (9)
C4B<2	20 (56)	16 (50)	8 (44)	20 (45)	0 (0)	5 (100)	24 (49)	14 (48)	6 (40)	6 (40)	7 (41)	9 (56)	5 (45)
C4A or C4B<2	29 (81)^b^	26 (81)	11(61)	31(70)^c^	0 (0)	5 (100)	35 (71)	22 (76)	10 (67)	11 (73)^d^	10 (59)	11 (69)	6 (55)

Values are expressed as number (%) of patients, unless otherwise stated.

a Underlying diseases classified according to the criteria of McCabe classification: 1) healthy i.e. no other diseases 2) non-fatal chronic diseases 3) ultimately fatal diseases with expected life expectancy of maximally 5 years 4) rapidly fatal diseases with expected survival for no more than 6 months.

b p = 0.042.

c Two female patients had both C4A<2 and C4B<2 deficiency and one female had a total *C4A* deficiency.

d One female patient had both C4A<2 and C4B<2 deficiency.

There were some differences in the phenotype frequencies of the C4 allotypes between the patient groups (Table 5). The A3, Q0 allotype was found in (12/50, 24%) of the NTM patients, which reflected the increased frequency of C4A deficiency in NTM patients. The C4 allotype, A3, 3 was less common in the NTM patients (11/50, 22%) than in the healthy controls (58/149 [39%], p = 0.030, Table 5). The allotype B1, 2 was found in only 6% of the NTM patients (3/50) but in 20% of the healthy controls (30/149), p = 0.020, Table 5).

**Table 5:** The most common C4 phenotypes in patients infected with a non-tuberculous mycobacteria (NTM) and Healthy group (H).

	NTM		H		p^b^
	n = 50^a^		n = 149		
	n	(%)	n	(%)	
C4A phenotype^c^					
3	12	(24)	21	(14)	0.103
3,2	3	(6)	9	(6)	1.000^d^
3,3	11	(22)	58	(39)	0.030
3,3,2	8	(16)	18	(12)	0.477
3,3,3	7	(14)	17	(11)	0.626
C4B phenotype					
0	6	(12)	15	(10)	0.700
1	15	(30)	37	(25)	0.464
1,1	16	(32)	52	(35)	0.708
1,2	3	(6)	30	(20)	0.020
1,3	2	(4)	3	(2)	0.601^d^
2	3	(6)	6	(4)	0.694^d^
2,2	0	(0)	5	(3)	0.333^d^

a Values are expressed as numbers (%) of patients with valid information unless otherwise stated.

b p Chi-squared test.

c One patient with C4A total deficiency.

d Fisher ' s exact test.

## Discussion

Our study suggests that deficiency of *C4* might have a role for susceptibility to a NTM infection. This finding is consistent with previous studies, which had revealed increased frequencies of complement *C4A* deficiency in mycobacterial infections, especially in patients with pulmonary MTB [22], [36]. To the best of our knowledge, ours is the first study to report *C4* deficiency at gene level in NTM infected patients. We observed that the deficiencies of *C4* in NTM infected patients were significantly more common (72%) than in the healthy controls (56%). Furthermore, our study pinpointed the high 81% frequency of the *C4* deficiency among female NTM patients, suggesting that *C4* deficiency might be a risk factor for this specific patient group for NTM infection.

The polymorphisms of the complement and its receptor genes have been associated as a susceptibility factor for pulmonary tuberculosis [19]. The defects of the complement will lead to production of quantitatively less active complement proteins, which might thus hamper the opsonization and phagosytosis [28]. The thick capsular lipid layer of the opportunistic NTM may be one important mechanism how NTM evade the host defense [29]. Moreover it has been hypothesized, that complement proteins are involved in a mechanism through which mycobacteria enter the cell and may thereby evade the adaptive immune response [30]. *C4* deficiency found in our study might not only limit the complement activation and interfere opsonisation but also affect the positive feedback of IL-12/IFN- γ in macrophages.

Complement C4 deficiencies are common. In European decent North Americans, 13% lack one of the two C4A alleles and 18% are deficient of one of the two C4B alleles [37]. C4 deficiencies are even more common in the Finnish population. In Finland, 17% lack one of the two C4A alleles and 38% lack one of the two C4B alleles [33]. In our study the A3, Q0 allotype reflecting *C4A* deficiency, had an increased occurrence among the female NTM patients. Deficiency in *C4A* has previously been associated to several autoimmune diseases and in adults with upper respiratory infections and pulmonary tuberculosis [22], [33]. In the recent report, *C4A* deficiency was suggested to predispose children and adolescents to recurrent respiratory infections. Also in this report girls predominated in patients with *C4A* deficiency [38] corroborating our results in NTM. Other studies have reported associations between *C4B* deficiency and another mycobacterial disease, leprosy [39]. In our study all the five patients with cutaneous NTM infection had *C4B* deficiency.

The *C4* deficiencies are associated with certain HLA alleles and are inherited together as haplotypes. The haplotype and allele frequencies differ significantly between populations and ethnicities [22], [23], [24], [37]. Yet, a *C4* deficiency might serve as a surrogate or a marker of an immunological deficit that is associated with the NTM infection because of the high linkage disequilibrium between genes of the MHC haplotypes. The analyses of the whole MHC haplotypes with landmark markers of several genes including complement *C4* and TNF in addition to the conventional HLA alleles are highly warranted in MHC associated disease studies such as NTM in different ethnicities.

NTM infections without previous underlying diseases usually have a high prevalence especially in elderly women [2], [3], [8], [40], [41]. This is probably due to influence of aging on the immune system, which might manifest in both cellular and humoral immune responses. Those changes might predispose postmenopausal women to autoimmune diseases and the NTM infections [13], [42]. Moreover, the average age of diagnosis of the NTM infection in our present study was 65 years and most of the patients (64%) were otherwise healthy postmenopausal women. Elderly, slender female patients with the NTM infection often have bronchiectasis [3], [10], [43]. In this study 73% of the NTM patients with bronchiectasis had a deficiency in *C4*. Our results suggest that the *C4* deficiency and bronchiectasis might be one predisposing factor for pulmonary NTM infection especially in elderly women.

Although our patient material was small the advantage of this study was that NTM patients were of European descent without different ethnicities. Namely, the susceptibility to mycobacteria disease is variable among different ethnic groups (23, 24, 25, 26).

Our results must be regarded as preliminary due to small patient material although a C4 deficiency was statistically significantly more common in NTM patients as compared to healthy controls and even more clearly in female patients. On the other hand, the small sample size may have resulted that some clinically important (OR≥2.00) differences failed to reach significance. In the subgroup of women, the odds ratios of 2.15 and 2.04 failed to reach significance (Table 3). The power to find a significant difference in these analyses was only 33% and 37%, respectively and the required number of NTM patients were 102 and 87 subjects, respectively (with 0.05 two-sided significance level, 80% power and with unequal sample sizes; 1 patient:3 controls). The required total number of women was then 407 and 346 to observe a real difference in C4A and C4B in female populations, respectively. Due to the sample size limitations, the results of the subgroup analyses should be regarded as tentative.

The environmental risk factors with distinct associations to NTM disease are controversial [44], [45] and environmental effects together with genetic default should be focused on more specifically when studying pulmonary NTM disease. In this study more than half of the NTM patients (27/50) had never smoked and 25 of these non-smokers were female. There were 23 current or ex-smokers, but only 11 of them were female.

In conclusion, our study indicates that Finnish NTM patients had significantly more often *C4* deficiencies than the healthy control subjects. The findings of this study suggest that both a deficiency of complement *C4* and bronchiectasis in healthy females might have a role as risk factors for pulmonary NTM infections. Due to the sample size limitations, the results of this preliminary study should be regarded as tentative. Further investigation with a larger study to confirm the current findings is obvious.
