# Correction: Complement C4 Deficiency – A Plausible Risk Factor for Non-Tuberculous Mycobacteria (NTM) Infection in Apparently Immunocompetent Patients

**DOI:** 10.1371/journal.pone.0100656

**Published:** 2014-06-16

**Authors:** The PLOS ONE Staff

There is an error in the Funding statement for this article that was introduced during the preparation of this manuscript for publication. The correct Funding statement is:

“This study was financially supported by a research grant from the Finnish Lung Health Association (FILHA) and City of Helsinki, Department of Social Services and Health Care, which are gratefully acknowledged. No commercial sponsors took part in the funding. The funders had no role in study design, data collection and analysis, decision to publish, or preparation of the manuscript.”

In addition, the PDF version of this article does not contain all the 45 references for this paper. Please refer to the online version to obtain the full list of references.
